# Interesting to Graduates of the Baltimore College of Dental Surgery

**Published:** 1867-11

**Authors:** 


*Interesting to Graduates of the Baltimore College of Dental Surgery.*— We are pleased to announce that *Graduates of the Baltimore College of Dental Surgery* have the privilege of presenting themselves as candidates for the degree of Doctor of Medicine on attending *one session* of Medical Lectures in the Washington University School of Medicine of this city. In other words a diploma from the Baltimore Dental College is considered equivalent to one course of medical lectures in this Medical College. From the well-known ability and high standing in the medical profession of the gentlemen composing the Faculty of the Washington University, a faculty second to none in this country, we regard the above action as quite a compliment to the Baltimore College of Dental Surgery, and we know that many of the Alumni of the Baltimore Dental College will avail themselves of this opportunity for obtaining the degree of Doctor of Medicine.

